# Theory and performance of substitution models for estimating relative causal effects in nutritional epidemiology

**DOI:** 10.1093/ajcn/nqac188

**Published:** 2022-10-13

**Authors:** Georgia D Tomova, Mark S Gilthorpe, Peter W G Tennant

**Affiliations:** Leeds Institute for Data Analytics, University of Leeds, Leeds, United Kingdom; Faculty of Medicine and Health, University of Leeds, Leeds, United Kingdom; The Alan Turing Institute, London, United Kingdom; Leeds Institute for Data Analytics, University of Leeds, Leeds, United Kingdom; The Alan Turing Institute, London, United Kingdom; Obesity Institute, Leeds Beckett University, Leeds, United Kingdom; Leeds Institute for Data Analytics, University of Leeds, Leeds, United Kingdom; Faculty of Medicine and Health, University of Leeds, Leeds, United Kingdom; The Alan Turing Institute, London, United Kingdom

**Keywords:** nutritional epidemiology, substitution models, substitution analysis, estimand, causal inference, compositional data

## Abstract

**Background:**

Estimating relative causal effects (i.e., “substitution effects”) is a common aim of nutritional research. In observational data, this is usually attempted using 1 of 2 statistical modeling approaches: the leave-one-out model and the energy partition model. Despite their widespread use, there are concerns that neither approach is well understood in practice.

**Objectives:**

We aimed to explore and illustrate the theory and performance of the leave-one-out and energy partition models for estimating substitution effects in nutritional epidemiology.

**Methods:**

Monte Carlo data simulations were used to illustrate the theory and performance of both the leave-one-out model and energy partition model, by considering 3 broad types of causal effect estimands: *1*) direct substitutions of the exposure with a single component, *2*) inadvertent substitutions of the exposure with several components, and *3*) average relative causal effects of the exposure instead of all other dietary sources. Models containing macronutrients, foods measured in calories, and foods measured in grams were all examined.

**Results:**

The leave-one-out and energy partition models both performed equally well when the target estimand involved substituting a single exposure with a single component, provided all variables were measured in the same units. Bias occurred when the substitution involved >1 substituting component. Leave-one-out models that examined foods in mass while adjusting for total energy intake evaluated obscure estimands.

**Conclusions:**

Regardless of the approach, substitution models need to be constructed from clearly defined causal effect estimands. Estimands involving a single exposure and a single substituting component are typically estimated more accurately than estimands involving more complex substitutions. The practice of examining foods measured in grams or portions while adjusting for total energy intake is likely to deliver obscure relative effect estimands with unclear interpretations.

See corresponding editorial on page 1195.

## Introduction

Dietary guidelines often recommend substituting certain nutrients or foods with healthier alternatives. For instance, the United Kingdom's Scientific Advisory Committee on Nutrition recommends substituting saturated fats with unsaturated fats, to help ensure that saturated fats do not contribute >10% to total energy (1). Other examples include recommendations to replace refined grains with whole grains (2), and sugars with complex carbohydrates (3). Substitutions like these are typically informed by the cumulative evidence from both randomized control trials and prospective cohort studies.

In experimental studies, food substitutions are examined by conducting isocaloric dietary interventions (4). In contrast, observational studies must rely on estimating substitution effects using statistical methods and 2 approaches have become common. The first, known as the leave-one-out model, is a variation of the “standard model” for energy intake adjustment (5). Conceived as a means to emulate isocaloric interventions, this approach involves adjusting for total energy intake and all dietary sources except those being substituted with the exposure. The second approach, known as the energy partition model, involves including all sources of total energy intake as model covariates and subsequently subtracting the regression coefficients to estimate the substitution (6, 7).

Despite many previous attempts to explain the theory of substitution modeling in nutritional epidemiology (5, 8–11), and although both the leave-one-out and energy partition models are common in practice (5), there are concerns that they are not well understood (5, 9, 10). This study hence aims to introduce, examine, and illustrate the theory and performance of the different substitution modeling approaches in nutritional epidemiology using a causal inference perspective and a series of illustrative simulations.

### Target estimands and modeling approaches

Although the interpretation of substitution models has been discussed extensively elsewhere (5, 8–11), none have formally defined the causal effect estimands that these models target. When a substitution is sought in compositional data, i.e., data where ≥2 component “part” variables sum to a total “whole” variable (12), the corresponding target estimand represents a type of relative causal effect (13). Substitution models thus aim to estimate the joint effects of increasing the intake of an exposure while simultaneously decreasing the intake of ≥1 substituting components to maintain the same total (14, 15).

Depending on their specification, both the leave-one-out model and energy partition model can be used to estimate a range of relative effect estimands.

#### The leave-one-out model

Because the leave-one-out model adjusts for total energy intake, and total energy intake is a collider for all sources of energy intake, it introduces a dependency (i.e., substituting effect) between all sources of energy intake that are not controlled in addition (13, 14). If adjustment is made only for the exposure and total energy intake, then the model estimates the average relative causal effect (i.e., the relative causal effect of the exposure instead of all other components) because all other components are free to contribute to keeping the total fixed (14, 15). For more specific substitutions, ≥1 components may be adjusted in addition to prevent them from contributing, thereby removing them from the substitution. Typically, a single component is left unadjusted (i.e., “left out” of the model) to estimate the relative causal effect of ${X_1}$ instead of ${X_2}$, where ${X_1}$ is the exposure and ${X_2}$ is the excluded component, giving the model its name (5). Substitutions between the exposure and >1 component may also be estimated by leaving ≥2 components unadjusted.

The leave-one-out model may be used to estimate isocaloric substitution effects for both macronutrients and foods, providing all components are measured in calories and sum to the total energy intake. Where foods are measured in grams or portions, they will not be compositional with total energy. Equal-mass substitution effects may instead be estimated by adjusting for the total food intake in grams or portions, respectively, but “mixed unit” models that adjust for total energy while examining foods in grams or portions will evaluate obscure estimands. Unfortunately, it is common for investigators to adjust for total energy intake without understanding the estimands that are produced (10, 14).

In practice, most leave-one-out models may be inadvertent, i.e., built without the specific intention of estimating a relative causal effect. Adjustment for total energy intake is practiced for a variety of reasons (8), without full appreciation of the impact on the estimand. For example, it is common for researchers to adjust for total energy alongside a selection of macronutrients or foods deemed “important confounders” without reference to a target substitution estimand (16–20).

#### The energy partition model and all-components model

Although mathematically similar to the leave-one-out model, the energy partition model is philosophically distinct because it does not directly output substitution effects (7, 21). Instead, total energy intake is partitioned into constituent components, which are included as model covariates. The relative causal effect of ${X_1}$ instead of ${X_2}$ is then estimated from the difference between the β-coefficients for the exposure and the substituting component. Provided both components are measured in calories, this will be an isocaloric substitution. Alternatively, the average relative causal effect may be estimated by including and comparing terms for the exposure and a summary term for all “remaining energy intake” (i.e., the total energy intake from all components except the exposure). Bespoke substitutions can also be obtained by retaining all components, and explicitly calculating and comparing the weighted effects. For example, the average relative causal effect could be calculated from a model with all components by comparing the coefficient of the exposure with a weighted sum of the coefficients for all other components. Although technically a form of the energy partition model, we have previously named this latter approach the all-components model to differentiate it from other forms of the energy partition model (14). Like the leave-one-out model, the energy partition model can also be used with foods in grams to estimate equal-mass substitutions, provided the exposure and substituting components are all measured in grams.

## Methods

### Illustrative example

To illustrate the theory and performance of each approach in estimating different relative (substitution) causal effects, we considered the effects of a macronutrient exposure, nonmilk extrinsic sugars (or “sugars”; kcal), and a food exposure, meat (kcal and g), on fasting plasma glucose concentration (FPG). The choice of exposures and outcome was arbitrary, and the presented causal effects were chosen for illustration.

### Data simulation

**FIGURE 1:**
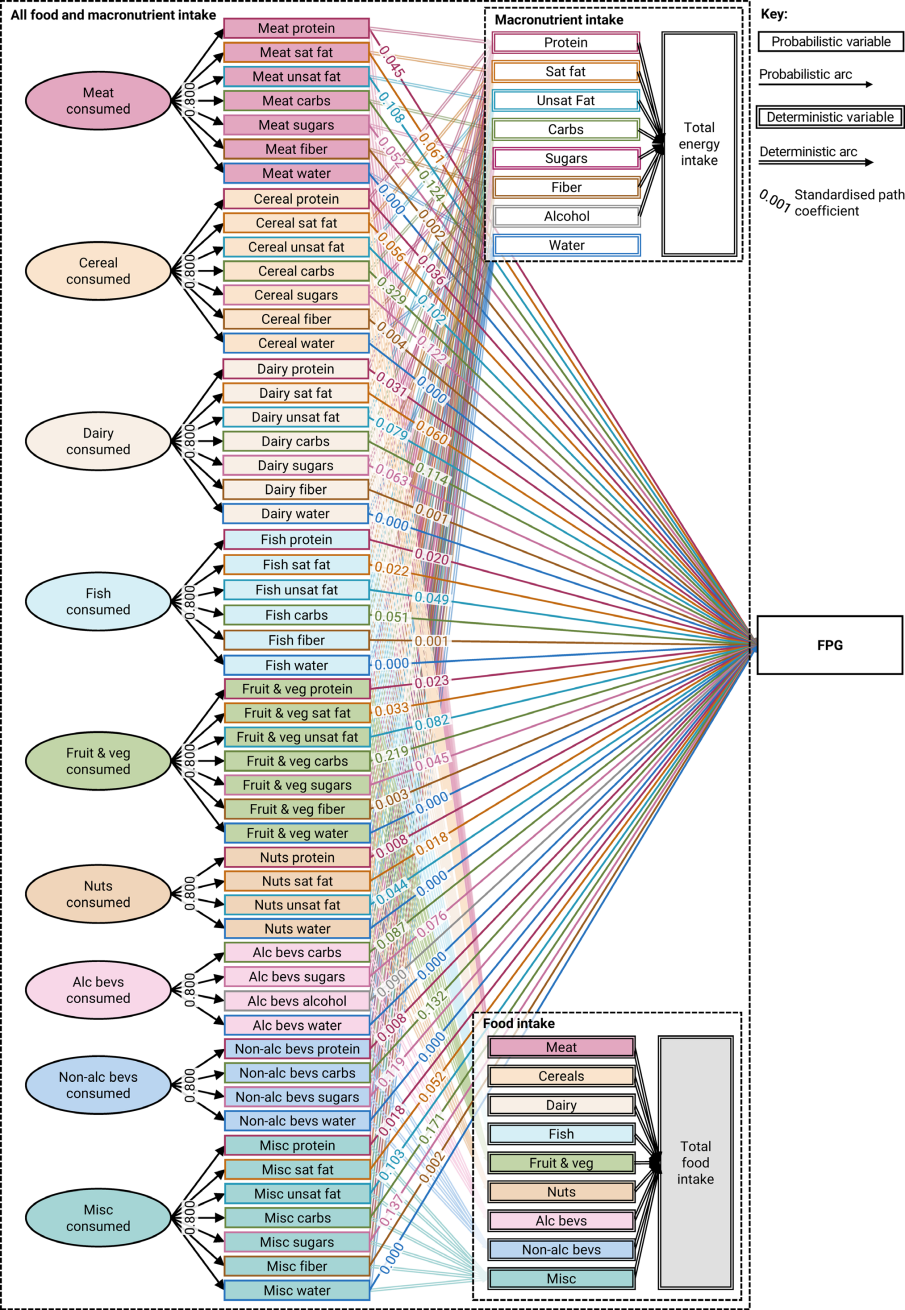
Parametric directed acyclic graph of the simulated data generating process, showing the standardized path coefficients used in the simulation. This directed acyclic graph uses the notation introduced in Arnold et al. (13), which depicts deterministic variables and deterministic relations using double-outlined rectangles and double-lined arrows, respectively. Deterministic variables are variables that are fully determined by their parent variables, e.g., total energy intake is fully determined by the energy intake from the 7 macronutrients, which are in turn fully determined by the energy intake from the 45 component variables. All deterministic variables were derived from their parent variables; the path coefficients for deterministic relations are therefore not shown. FPG, fasting plasma glucose.

Standardized data were simulated with the R package “dagitty” version 0.3-1 (22) based on a prespecified data generation process depicted in **Figure 1**. The simulated data reflect a scenario in which total energy intake is fully determined by the energy intake from 7 macronutrients and total food intake is fully determined by the intake from 9 food groups. The 7 macronutrients were *1*) sugars, *2*) complex carbohydrates, *3*) fiber, *4*) saturated fat, *5*) unsaturated fat, *6*) protein, and *7*) alcohol; and the 9 food groups were *1*) cereals, *2*) dairy, *3*) meat, *4*) fish, *5*) fruits and vegetables, *6*) nuts, *7*) alcoholic beverages, *8*) nonalcoholic beverages, and *9*) miscellaneous. Each macronutrient was assigned a unique effect on FPG using different path coefficients. Target means and SDs for each macronutrient (kcal) were informed by the United Kingdom's National Diet and Nutrition Survey (23), with SDs capped to ensure convergence (see **Supplemental Table 1**). Component macronutrient variables, and component water variables, were then simulated for each food and their means and SDs were scaled to match the distribution observed in the National Diet and Nutrition Survey (**Supplemental Table 2**) and the target means and SDs for the total intake of each macronutrient (Supplemental Table 1). Food intakes in calories were derived by summing the constituent macronutrients, and food intakes in grams were derived by adding the constituent macronutrients multiplied by their caloric density, with water added to match the moisture content observed in the National Diet and Nutrition Survey. Total energy intake (i.e., total calories consumed from all dietary sources), total food intake (i.e., total mass of food consumed from all dietary sources), remaining energy intake (i.e., total calories consumed from all dietary sources except the exposure), and remaining food intake (i.e., total food consumed from all dietary sources except the exposure) were calculated directly from the simulated variables to reflect the deterministic data generation process. In total, 100,000 simulated data sets were created, each comprising 1000 observations. We report the median effect estimate and the 2.5th and 97.5th centiles [representing 95% simulation interval (SI)] for each investigated model. To aid illustration, effect estimates are expressed in mg/dL per 100 kcal of nutrients or foods consumed (i.e., mg · dL^−1^ · 100 kcal^−1^) and mg/dL per 100 g of foods consumed (i.e., mg · dL^−1^ · 100 g^−1^).

### Estimands and models examined

We examined 3 types of causal effect estimands across the 3 dietary unit scenarios of macronutrients (kcal), foods (kcal), and foods (g). The first group of estimands involved a direct substitution of the exposure for a single component. For macronutrients this was the relative causal effect of sugars instead of protein (${R_1}$), and for foods this was the relative causal effect of meat instead of fish (kcal, ${R_4}$; and g, ${R_7}$). The second group of estimands involved a more complex substitution of the exposure for several components, as might arise from inadvertently adjusting for total energy intake without specifically intending to estimate a relative causal effect. For macronutrients, we considered the (inadvertent) estimand created by a model that adjusted for carbohydrates, alcohol, and total energy viewed as “important confounders”; this was the (inadvertent) relative causal effect of sugars instead of protein, total fat, and fiber (${R_2}$). For foods, we considered the (inadvertent) estimands created by models that adjusted for fruit and veg, alcoholic beverages, nonalcoholic beverages, and total energy or total food; these were the (inadvertent) relative causal effects of meat instead of cereal, dairy, fish, nuts, and miscellaneous food in calories (${R_5}$) and grams (${R_8}$). The third group of estimands involved a substitution of the exposure for all other dietary components, i.e., average relative causal effects. For macronutrients this was the average relative causal effect of sugars (${R_3}$) and for foods this was the average relative causal effect of meat (kcal, ${R_6}$; and g, ${R_9}$). Each estimand was evaluated using a “same unit” leave-one-out model, a comprehensive energy partition (i.e., all-components) model, and, where relevant, a simplified energy partition model and a “mixed unit” leave-one-out model. “Same unit” leave-one-out models are isocaloric and equal-mass models where the components are measured in calories and grams, respectively, and adjustment is made for total energy intake and total food intake, respectively. “Mixed unit” leave-one-out models are obscure models where the components are measured in grams and adjustment is made for total energy intake. We compared the estimates obtained from each model either with the simulated truth (for the macronutrient models) or with estimates derived from the weighted average of the macronutrients making up each food (for the food group models). The **Supplemental Methods** provide further details of the estimands and models.

## Results

**Table 1** summarizes the results of the 24 models.

**TABLE 1:** Full details of the 9 estimands and 24 models examined in the simulated data^1^

Estimand			Model				
Estimand symbol	Name	Simulated or derived^2^ value	Estimate symbol	Name	Formula		Estimated value (95% SI)
${R_1}$	Relative causal effect of sugars instead of protein	3.20^3^	$\widehat {{R_{1.1}}}$	“Same unit” leave-one-out	$\widehat {{\rm{FPG}}} = $	$\widehat {{a_0}} + \widehat {{a_1}}{\rm{sugars}} + \,\,\widehat {{a_2}}{\rm{carbs}} + \widehat {{a_3}}{\rm{unsat\,\,fat}} + \widehat {{a_4}}{\rm{sat\,\,fat}} + \widehat {{a_5}}{\rm{fiber}} + \widehat {{a_6}}{\rm{alcohol}} + \widehat {{a_7}}{\rm{TE}} + \varepsilon $	
					$\widehat {{R_{1.1}}} = $	$\widehat {{{\rm{a}}_1}}$	3.20^3^ (2.52, 3.88)
			$\widehat {{R_{1.2}}}$	(Comprehensive) energy partition/all-components	$\widehat {{\rm{FPG}}} = $	$\widehat {{{\rm{b}}_0}} + \widehat {{{\rm{b}}_1}}{\rm{sugars}} + \widehat {{{\rm{b}}_2}}{\rm{protein}} + \widehat {{{\rm{b}}_3}}{\rm{carbs}} + \widehat {{{\rm{b}}_4}}{\rm{unsat\,\,fat}} + \widehat {{{\rm{b}}_5}}{\rm{sat\,\,fat}} + \widehat {{{\rm{b}}_6}}{\rm{fiber}} + \,\,\widehat {{{\rm{b}}_7}}{\rm{alcohol}} + \varepsilon $	
					$\widehat {{R_{1.2}}} = $	$\widehat {{{\rm{b}}_1}} - \,\,\widehat {{{\rm{b}}_2}}$	3.20^3^ (2.52, 3.88)
${R_2}$	“Inadvertent” relative causal effect of sugars instead of protein, total fat, and fiber	2.50^3^	$\widehat {{R_{2.1}}}$	“Same unit” leave-one-out	$\widehat {{\rm{FPG}}} = $	$\widehat {{{\rm{c}}_0}} + \widehat {{{\rm{c}}_1}}{\rm{sugars}} + \widehat {{{\rm{c}}_2}}{\rm{TE}} + \widehat {{{\rm{c}}_3}}{\rm{carbs}} + \widehat {{{\rm{c}}_4}}{\rm{alcohol}} + \varepsilon $	
					$\widehat {{R_{2.1}}} = $	$\widehat {{c_1}}$	2.39^3^ (1.92, 2.85)
			$\widehat {{R_{2.2}}}$	All-components	$\widehat {{\rm{FPG}}} = $	$\widehat {{{\rm{b}}_0}} + \widehat {{{\rm{b}}_1}}{\rm{sugars}} + \widehat {{{\rm{b}}_2}}{\rm{protein}} + \widehat {{{\rm{b}}_3}}{\rm{carbs}} + \widehat {{{\rm{b}}_4}}{\rm{unsat\,\,fat}} + \widehat {{{\rm{b}}_5}}{\rm{sat\,\,fat}} + \widehat {{{\rm{b}}_6}}{\rm{fiber}} + \,\,\widehat {{{\rm{b}}_7}}{\rm{alcohol}} + \varepsilon $	
					$\widehat {{R_{2.2}}} = $	$\widehat {{b_1}} - \widehat {{b_{2,4,5,6}}}$ where $\widehat {{b_{2,4,5,6}}} = \sum i{w_i}{b_i}$ and $i = \{ {2,4,5,6} \}$	2.50^3^ (2.02, 2.98)
${R_3}$	Average relative causal effect of sugar	1.75^3^	$\widehat {{R_{3.1}}}$	“Same unit” leave-one-out	$\widehat {{\rm{FPG}}} = $	${\rm{\,\,}}\widehat {{{\rm{d}}_0}} + \widehat {{{\rm{d}}_1}}{\rm{sugars}} + \widehat {{{\rm{d}}_2}}{\rm{TE}} + \varepsilon $	
					$\widehat {{R_{3.1}}} = $	$\widehat {{d_1}}$	2.05^3^ (1.57, 2.52)
			$\widehat {{R_{3.2}}}$	(Simple) energy partition	$\widehat {{\rm{FPG}}} = $	$\widehat {{{\rm{f}}_0}} + \widehat {{{\rm{f}}_1}}{\rm{sugars}} + \widehat {{{\rm{f}}_2}}{\rm{RE}} + \varepsilon $	
					$\widehat {{R_{3.2}}} = $	$\widehat {{{\rm{f}}_1}} - \,\,\widehat {{{\rm{f}}_2}}$	2.05^3^ (1.57, 2.52)
			$\widehat {{R_{3.3}}}$	(Comprehensive) energy partition/all-components	$\widehat {{\rm{FPG}}} = $	$\widehat {{{\rm{b}}_0}} + \widehat {{{\rm{b}}_1}}{\rm{sugars}} + \widehat {{{\rm{b}}_2}}{\rm{protein}} + \widehat {{{\rm{b}}_3}}{\rm{carbs}} + \widehat {{{\rm{b}}_4}}{\rm{unsat\,\,fat}} + \widehat {{{\rm{b}}_5}}{\rm{sat\,\,fat}} + \widehat {{{\rm{b}}_6}}{\rm{fiber}} + \,\,\widehat {{{\rm{b}}_7}}{\rm{alcohol}} + \varepsilon $	
					$\widehat {{R_{3.3}}} = $	$\widehat {{b_1}} - \widehat {{b_{2:7}}}$ where $\widehat {{b_{2:7}}} = \sum i{w_i}{b_i}$ and $i = \{ {2,3,4,5,6,7} \}$	1.75^3^ (1.29, 2.21)
${R_4}$	Relative causal effect of meat instead of fish (kcal)	0.16^3^	$\widehat {{R_{4.1}}}$	“Same unit” leave-one-out	$\widehat {{\rm{FPG}}} = $	$\widehat {{{\rm{g}}_0}} + \widehat {{{\rm{g}}_1}}{\rm{meat}} + \widehat {{{\rm{g}}_2}}{\rm{cereal}} + \widehat {{{\rm{g}}_3}}{\rm{dairy}} + \widehat {{{\rm{g}}_4}}{\rm{fruit\,\,veg}} + \widehat {{{\rm{g}}_5}}{\rm{nuts}} + \widehat {{{\rm{g}}_6}}{\rm{alc\,\,bev}} + \,\,\widehat {{{\rm{g}}_7}}{\rm{nonalc\,\,bev}} + \,\,\widehat {{{\rm{g}}_8}}{\rm{misc}} + \widehat {{{\rm{g}}_9}}{\rm{TE}} + \varepsilon $	
					$\widehat {{R_{4.1}}} = $	$\widehat {{g_1}}$	0.20^3^ (−0.21, 0.61)
			$\widehat {{R_{4.2}}}$	(Comprehensive) energy partition/all-components	$\widehat {{\rm{FPG}}} = $	$\widehat {{{\rm{h}}_0}} + \widehat {{{\rm{h}}_1}}{\rm{meat}} + \widehat {{{\rm{h}}_2}}{\rm{cereal}} + \widehat {{{\rm{h}}_3}}{\rm{dairy}} + \widehat {{{\rm{h}}_4}}{\rm{fish}} + \widehat {{{\rm{h}}_5}}{\rm{fruit\,\,veg}} + \widehat {{{\rm{h}}_6}}{\rm{nuts}} + \,\,\widehat {{{\rm{h}}_7}}{\rm{alc\,\,bev}} + \,\,\widehat {{{\rm{h}}_8}}{\rm{nonalc\,\,bev}} + \widehat {{{\rm{h}}_9}}{\rm{misc}} + \varepsilon $	
					$\widehat {{R_{4.2}}} = $	$\widehat {{{\rm{h}}_1}} - \,\,\widehat {{{\rm{h}}_4}}$	0.20^3^ (−0.21, 0.61)
${R_5}$	“Inadvertent” relative causal effect of meat instead of cereal, dairy, fish, nuts, and misc. (kcal)	−0.65^3^	$\widehat {{R_{5.1}}}$	(Inadvertent) “same unit” leave-one-out	$\widehat {{\rm{FPG}}} = $	$\widehat {{{\rm{i}}_0}} + \widehat {{{\rm{i}}_1}}{\rm{meat}} + \widehat {{{\rm{i}}_2}}{\rm{fruit\,\,veg}} + \widehat {{{\rm{i}}_3}}{\rm{alc\,\,bev}} + \widehat {{{\rm{i}}_4}}{\rm{nonalc\,\,bev}} + \widehat {{{\rm{i}}_5}}{\rm{TE}} + \varepsilon $	
					$\widehat {{R_{5.1}}} = $	$\widehat {{i_1}}$	−0.37^3^ (−0.52, −0.20)
			$\widehat {{R_{5.2}}}$	All-components	$\widehat {{\rm{FPG}}} = $	$\widehat {{{\rm{h}}_0}} + \widehat {{{\rm{h}}_1}}{\rm{meat}} + \widehat {{{\rm{h}}_2}}{\rm{cereal}} + \widehat {{{\rm{h}}_3}}{\rm{dairy}} + \widehat {{{\rm{h}}_4}}{\rm{fish}} + \widehat {{{\rm{h}}_5}}{\rm{fruit\,\,veg}} + \widehat {{{\rm{h}}_6}}{\rm{nuts}} + \,\,\widehat {{{\rm{h}}_7}}{\rm{alc\,\,bev}} + \,\,\widehat {{{\rm{h}}_8}}{\rm{nonalc\,\,bev}} + \widehat {{{\rm{h}}_9}}{\rm{misc}} + \varepsilon $	
					$\widehat {{R_{5.2}}} = $	$\widehat {{h_1}} - \widehat {{h_{2,3,4,6,9}}}$ where $\widehat {{h_{2,3,4,6,9}}} = \sum i{w_i}{h_i}$ and $i = \{ {2,3,4,6,9} \}$	−0.35^3^ (−0.52, −0.19)
${R_6}$	Average relative causal effect of meat (kcal)	−0.78^3^	$\widehat {{R_{6.1}}}$	“Same unit” leave-one-out	$\widehat {{\rm{FPG}}} = $	$\widehat {{{\rm{j}}_0}} + \widehat {{{\rm{j}}_1}}{\rm{meat}} + \widehat {{{\rm{j}}_2}}{\rm{TE}} + \varepsilon $	
					$\widehat {{R_{6.1}}} = $	$\widehat {{j_1}}$	−0.42^3^ (−0.59, −0.25)
			$\widehat {{R_{6.2}}}$	(Simple) energy partition	$\widehat {{\rm{FPG}}} = $	$\widehat {{{\rm{k}}_0}} + \widehat {{{\rm{k}}_1}}{\rm{meat}} + \widehat {{{\rm{k}}_2}}{\rm{RE}} + \varepsilon $	
					$\widehat {{R_{6.2}}} = $	$\widehat {{{\rm{k}}_1}} - \,\,\widehat {{{\rm{k}}_2}}$	−0.42^3^ (−0.59, −0.25)
			$\widehat {{R_{6.3}}}$	(Comprehensive) energy partition/all-components	$\widehat {{\rm{FPG}}} = $	$\widehat {{{\rm{h}}_0}} + \widehat {{{\rm{h}}_1}}{\rm{meat}} + \widehat {{{\rm{h}}_2}}{\rm{cereal}} + \widehat {{{\rm{h}}_3}}{\rm{dairy}} + \widehat {{{\rm{h}}_4}}{\rm{fish}} + \widehat {{{\rm{h}}_5}}{\rm{fruit\,\,veg}} + \widehat {{{\rm{h}}_6}}{\rm{nuts}} + \,\,\widehat {{{\rm{h}}_7}}{\rm{alc\,\,bev}} + \,\,\widehat {{{\rm{h}}_8}}{\rm{nonalc\,\,bev}} + \widehat {{{\rm{h}}_9}}{\rm{misc}} + \varepsilon $	
					$\widehat {{R_{6.3}}} = $	$\widehat {{h_1}} - \widehat {{h_{2:9}}}$ where $\widehat {{h_{2:9}}} = \sum i{w_i}{h_i}$ and $i = \{ {2,3,4,5,6,7,8,9} \}$	−0.47^3^ (−0.63, −0.30)
${R_7}$	Relative causal effect of meat instead of fish (g)	5.15^4^	$\widehat {{R_{7.1}}}$	“Same unit” leave-one-out (with total food intake)	$\widehat {{\rm{FPG}}} = $	$\widehat {{{\rm{l}}_0}} + \widehat {{{\rm{l}}_1}}{\rm{meat}} + \widehat {{{\rm{l}}_2}}{\rm{cereal}} + \widehat {{{\rm{l}}_3}}{\rm{dairy}} + \widehat {{{\rm{l}}_4}}{\rm{fruit\,\,veg}} + \widehat {{{\rm{l}}_5}}{\rm{nuts}} + \widehat {{{\rm{l}}_6}}{\rm{alc\,\,bev}} + \,\,\widehat {{{\rm{l}}_7}}{\rm{nonalc\,\,bev}} + \,\,\widehat {{{\rm{l}}_8}}{\rm{misc}} + \widehat {{{\rm{l}}_9}}{\rm{TF}} + \varepsilon $	
					$\widehat {{R_{7.1}}} = $	$\widehat {{{\rm{l}}_1}}$	4.81^4^ (3.23, 6.32)
			$\widehat {{R_{7.2}}}$	“Mixed unit” leave-one-out (with total energy)	$\widehat {{\rm{FPG}}} = $	$\widehat {{{\rm{m}}_0}} + \widehat {{{\rm{m}}_1}}{\rm{meat}} + \widehat {{{\rm{m}}_2}}{\rm{cereal}} + \widehat {{{\rm{m}}_3}}{\rm{dairy}} + \widehat {{{\rm{m}}_4}}{\rm{fruit\,\,veg}} + \widehat {{{\rm{m}}_5}}{\rm{nuts}} + \widehat {{{\rm{m}}_6}}{\rm{alc\,\,bev}} + \,\,\widehat {{{\rm{m}}_7}}{\rm{nonalc\,\,bev}} + \,\,\widehat {{{\rm{m}}_8}}{\rm{misc}} + \widehat {{{\rm{m}}_9}}{\rm{TE}} + \varepsilon $	
					$\widehat {{R_{7.2}}} = $	$\widehat {{{\rm{m}}_1}}$	−0.94^4^ (−1.58, −0.31)
			$\widehat {{R_{7.3}}}$	All-components	$\widehat {{\rm{FPG}}} = $	$\widehat {{{\rm{n}}_0}} + \widehat {{{\rm{n}}_1}}{\rm{meat}} + \widehat {{{\rm{n}}_2}}{\rm{cereal}} + \widehat {{{\rm{n}}_3}}{\rm{dairy}} + \widehat {{{\rm{n}}_4}}{\rm{fish}} + \widehat {{{\rm{n}}_5}}{\rm{fruit\,\,veg}} + \widehat {{{\rm{n}}_6}}{\rm{nuts}} + \,\,\widehat {{{\rm{n}}_7}}{\rm{alc\,\,bev}} + \,\,\widehat {{{\rm{n}}_8}}{\rm{nonalc\,\,bev}} + \widehat {{{\rm{n}}_9}}{\rm{misc}} + \varepsilon $	
					$\widehat {{R_{7.3}}} = $	$\widehat {{{\rm{n}}_1}} - \widehat {{{\rm{n}}_4}}$	4.81^4^ (3.23, 6.32)
${R_8}$	“Inadvertent” relative causal effect of meat instead of cereal, dairy, fish, nuts, and misc. (g)	1.44^4^	$\widehat {{R_{8.1}}}$	(Inadvertent) “same unit” leave-one-out (with total food intake)	$\widehat {{\rm{FPG}}} = $	$\widehat {{{\rm{o}}_0}} + \widehat {{{\rm{o}}_1}}{\rm{meat}} + \widehat {{{\rm{o}}_2}}{\rm{fruit\,\,veg}} + \widehat {{{\rm{o}}_3}}{\rm{alc\,\,bev}} + \widehat {{{\rm{o}}_4}}{\rm{nonalc\,\,bev}} + \widehat {{{\rm{o}}_5}}{\rm{TF}} + \varepsilon $	
					$\widehat {{R_{8.1}}} = $	$\widehat {{{\rm{o}}_1}}$	4.60^4^ (3.21, 6.06)
			$\widehat {{R_{8.2}}}$	(Inadvertent) “mixed unit” leave-one-out (with total energy)	$\widehat {{\rm{FPG}}} = $	$\widehat {{{\rm{p}}_0}} + \widehat {{{\rm{p}}_1}}{\rm{meat}} + \widehat {{{\rm{p}}_2}}{\rm{fruit\,\,veg}} + \widehat {{{\rm{p}}_3}}{\rm{alc\,\,bev}} + \widehat {{{\rm{p}}_4}}{\rm{nonalc\,\,bev}} + \widehat {{{\rm{p}}_5}}{\rm{TE}} + \varepsilon $	
					$\widehat {{R_{8.2}}} = $	$\widehat {{{\rm{p}}_1}}$	−1.11^4^ (−1.64, −0.60)
			$\widehat {{R_{8.3}}}$	All-components	$\widehat {{\rm{FPG}}} = $	$\widehat {{{\rm{n}}_0}} + \widehat {{{\rm{n}}_1}}{\rm{meat}} + \widehat {{{\rm{n}}_2}}{\rm{cereal}} + \widehat {{{\rm{n}}_3}}{\rm{dairy}} + \widehat {{{\rm{n}}_4}}{\rm{fish}} + \widehat {{{\rm{n}}_5}}{\rm{fruit\,\,veg}} + \widehat {{{\rm{n}}_6}}{\rm{nuts}} + \,\,\widehat {{{\rm{n}}_7}}{\rm{alc\,\,bev}} + \,\,\widehat {{{\rm{n}}_8}}{\rm{nonalc\,\,bev}} + \widehat {{{\rm{n}}_9}}{\rm{misc}} + \varepsilon $	
					$\widehat {{R_{8.3}}} = $	$\widehat {{n_1}} - \widehat {{n_{2,3,4,6,9}}}$ where $\widehat {{n_{2,3,4,6,9}}} = \sum i{w_i}{n_i}$ and $i = \{ {2,3,4,6,9} \}$	5.42^4^ (4.51, 6.33)
${R_9}$	Average relative causal effect of meat (g)	6.48^4^	$\widehat {{R_{9.1}}}$	“Same unit” leave-one-out (with total food intake)	$\widehat {{\rm{FPG}}} = $	$\widehat {{{\rm{q}}_0}} + \widehat {{{\rm{q}}_1}}{\rm{meat}} + \widehat {{{\rm{q}}_2}}{\rm{TF}} + \varepsilon $	
					$\widehat {{R_{9.1}}} = $	$\widehat {{q_1}}$	8.14^4^ (6.55, 9.80)
			$\widehat {{R_{9.2}}}$	“Mixed unit” leave-one-out (with total energy)	$\widehat {{\rm{FPG}}} = $	$\widehat {{{\rm{r}}_0}} + \widehat {{{\rm{r}}_1}}{\rm{meat}} + \widehat {{{\rm{r}}_2}}{\rm{TE}} + \varepsilon $	
					$\widehat {{R_{9.2}}} = $	$\widehat {{{\rm{r}}_1}} - \,\,\widehat {{{\rm{r}}_2}}$	−1.17^4^ (−1.70, −0.66)
			$\widehat {{R_{9.3}}}$	(Simple) energy partition	$\widehat {{\rm{FPG}}} = $	$\widehat {{{\rm{s}}_0}} + \widehat {{{\rm{s}}_1}}{\rm{meat}} + \widehat {{{\rm{s}}_2}}{\rm{RF}} + \varepsilon $	
					$\widehat {{R_{9.3}}} = $	$\widehat {{{\rm{s}}_1}} - \,\,\widehat {{{\rm{s}}_2}}$	8.14^4^ (6.55, 9.80)
			$\widehat {{R_{9.4}}}$	All-components	$\widehat {{\rm{FPG}}} = $	$\widehat {{{\rm{n}}_0}} + \widehat {{{\rm{n}}_1}}{\rm{meat}} + \widehat {{{\rm{n}}_2}}{\rm{cereal}} + \widehat {{{\rm{n}}_3}}{\rm{dairy}} + \widehat {{{\rm{n}}_4}}{\rm{fish}} + \widehat {{{\rm{n}}_5}}{\rm{fruit\,\,veg}} + \widehat {{{\rm{n}}_6}}{\rm{nuts}} + \,\,\widehat {{{\rm{n}}_7}}{\rm{alc\,\,bev}} + \,\,\widehat {{{\rm{n}}_8}}{\rm{nonalc\,\,bev}} + \widehat {{{\rm{n}}_9}}{\rm{misc}} + \varepsilon $	
					$\widehat {{R_{9.4}}} = $	$\widehat {{n_1}} - \widehat {{n_{2:9}}}$ where $\widehat {{n_{2:9}}} = \sum i{w_i}{n_i}$ and $i = \{ {2,3,4,5,6,7,8,9} \}$	6.06^4^ (5.12, 7.05)

1 FPG, fasting plasma glucose; RE, remaining energy intake; RF, remaining food (mass) intake; SI, simulation interval; TE, total energy intake; TF, total food (mass) intake.

2 Estimated by directly calculating the weighted-average effect; for further details see the analytic code.

3 Units are mg · dL^−1^ · 100 kcal^−1^.

4 Units are mg · dL^−1^ · 100 g^−1^.

### Macronutrient models

Both the “same unit” leave-one-out and (comprehensive) energy partition (i.e., all-components) models returned the true causal effect when the substitution involved a single macronutrient ($\widehat {{R_{1.1}}}$ = $\widehat {{R_{1.2}}}$ = ${R_1}$ = 3.20 mg/dL/100 kcal, i.e., the causal effect of 100 kcal from sugar on FPG was estimated to be 3.20 mg/dL larger than the effect of 100 kcal coming from protein, for the same total energy intake).

Where the substitution involved >1 macronutrient, the all-components model again returned the expected true value ($\widehat {{R_{2.2}}}$ = ${R_2}$ = 2.50 mg/dL/100 kcal (i.e., the causal effect of 100 kcal from sugar on FPG was estimated to be 2.50 mg/dL larger than the average effect of 100 kcal coming from protein, fat, and fiber, for the same total energy intake), but the “same unit” leave-one-out model returned a slightly biased smaller value ($\widehat {{R_{2.1}}}$ = 2.39 mg/dL/100 kcal).

The all-components model returned the expected true value for the average relative causal effect of sugar ($\widehat {{R_{3.3}}}$ = ${R_3}$ = 1.75 mg/dL/100 kcal, i.e., the causal effect of 100 kcal from sugar on FPG was estimated to be 1.75 mg/dL larger than the average effect of 100 kcal coming from all other macronutrients, for the same total energy intake), but both the “same unit” leave-one-out model and the simple energy partition model returned the same slightly biased larger value ($\widehat {{R_{3.1}}}$ = $\widehat {{R_{3.2}}}$ = 2.05 mg/dL/100 kcal).

### Food group models—in calories

Both the “same unit” leave-one-out and (comprehensive) energy partition (i.e., all-components) models returned the same value when the substitution involved a single food ($\widehat {{R_{4.1}}}$ = $\widehat {{R_{4.2}}}$ = 0.20 mg/dL/100 kcal, i.e., the causal effect of 100 kcal from meat on FPG was estimated to be 0.20 mg/dL larger than the effect of 100 kcal coming from fish, for the same total energy intake), which was similar to but slightly larger than the expected true value (${R_4}$ = 0.16 mg/dL/100 kcal).

Where the substitution involved >1 variable, both the “same unit” leave-one-out and all-components models returned similar values ($\widehat {{R_{6.1}}}$ = −0.37 mg/dL/100 kcal and $\widehat {{R_{5.2}}}$ = −0.35 mg/dL/100 kcal, respectively, i.e., the causal effect of 100 kcal from meat on FPG was estimated to be 0.37 or 0.35 mg/dL smaller than the average effect of 100 kcal coming from cereal, dairy, fish, nuts, and miscellaneous foods, for the same total energy intake), both of which were smaller than and almost half the expected true value (${R_5}$ = −0.65 mg/dL/100 kcal).

The all-components model returned an average relative causal effect of meat ($\widehat {{R_{6.3}}}$ = −0.47 mg/dL/100 kcal, i.e., the causal effect of 100 kcal from meat on FPG was estimated to be 0.47 mg/dL smaller than the average effect of 100 kcal coming from all other foods, for the same total energy intake) that was smaller than and almost half the expected true value (${R_6}$ = −0.78 mg/dL/100 kcal). Both the “same unit” leave-one-out model and the simple energy partition model returned identical estimates that were slightly more biased ($\widehat {{R_{6.1}}}$ = $\widehat {{R_{6.2}}}$ = −0.42 mg/dL/100 kcal).

### Food group models—in grams

Both the “same unit” leave-one-out and (comprehensive) energy partition (i.e., all-components) models returned the same value when the substitution involved a single food and adjustment was made for total food intake ($\widehat {{R_{7.1}}}$ = $\widehat {{R_{7.3}}}$ = 4.81 mg/dL/100 g, i.e., the causal effect of 100 g from meat on FPG was estimated to be 4.81 mg/dL larger than the effect of 100 g coming from fish, for the same total food intake), which was slightly smaller than the expected true value (${R_7}$ = 5.15 mg/dL/100 g). The “mixed unit” leave-one-out-model that adjusted for total energy intake returned a radically different value with the opposite sign to both the isocaloric and equal-mass estimands ($\widehat {{R_{7.2}}}$ = −0.94 mg/dL/100 g, i.e., the causal effect of 100 g from meat on FPG was estimated to be 0.94 mg/dL smaller than the effect of 100 g coming from fish, for the same total energy intake).

Where the substitution involved >1 variable, both the “same unit” leave-one-out and all-components models returned similar values ($\widehat {{R_{8.1}}}$ = 4.60 mg/dL/100 g and $\widehat {{R_{8.3}}}$ = 5.42 mg/dL/100 g, respectively, i.e., the causal effect of 100 g from meat on FPG was estimated to be 4.60 or 5.42 mg/dL larger than the average effect of 100 g coming from cereal, dairy, fish, nuts, and miscellaneous foods, for the same total food intake), which were much larger than the derived estimate (${R_8}$ = 1.44 mg/dL/100 g). The “mixed unit” leave-one-out-model that adjusted for total energy intake returned a radically different value of equal sign to the isocaloric estimand and opposite sign to the equal-mass estimand ($\widehat {{R_{8.2}}}$ = −1.11 mg/dL/100 g, i.e., the causal effect of 100 g from meat on FPG was estimated to be 1.11 mg/dL smaller than the average effect of 100 g coming from cereal, dairy, fish, nuts, and miscellaneous foods, for the same total energy intake).

The all-components model returned a slightly smaller average relative causal effect of meat than the expected true value ($\widehat {{R_{9.4}}}$ = 6.06 mg/dL/100 g compared with ${R_9}$ = 6.48 mg/dL/100 g, i.e., the true causal effect of 100 g from meat on FPG was 6.48 mg/dL larger than the average effect of 100 g coming from all other foods, for the same total food intake). Both the “same unit” leave-one-out model and the simple energy partition model returned identical estimates that were larger than the expected true values ($\widehat {{R_{9.1}}}$ = $\widehat {{R_{9.3}}}$ = 8.14 mg/dL/100 g). The “mixed unit” leave-one-out-model that adjusted for total energy intake returned a radically different value of equal sign to the isocaloric estimand and opposite sign to the equal-mass estimand ($\widehat {{R_{9.2}}}$ = −1.17 mg/dL/100 g, i.e., the causal effect of 100 g from meat on FPG was estimated to be 1.17 mg/dL smaller than the average effect of 100 g coming from all other foods, for the same total energy intake).

## Discussion

### Principal findings

This study examined and compared the performance of the 2 most common approaches to estimating relative (i.e., substitution) effects in nutritional epidemiology. We demonstrated that “same unit” leave-one-out models and energy partition models perform equally well when the target estimand involves substituting a single exposure with a single component. Once the substitution involves ≥2 components, both models can produce biased estimates that diverge from the expected true weighted average effects.

We have also shown that isocaloric and equal-mass substitution estimates can be reliably estimated using either model provided all variables are measured in the same units. “Mixed unit” leave-one-out models that examine foods in mass while adjusting for total energy intake evaluate obscure estimands that may be sign discordant from both the isocaloric and equal-mass estimands.

### Analysis and explanation

In substitution modeling, the estimand is determined jointly by the exposure and by the dietary components chosen for substitution. There is hence no single “relative causal effect” but an array of potential estimands that may be considered, each requiring appropriate modeling and interpretation. Because nutritional data are compositional, the causal effect being estimated by a particular model may depend not only on the components in the model, but also on those that are absent, whether purposefully or inadvertently. This is particularly true where the “total” is conditioned on, because a relative effect will be established with all components that are absent from the model (13).

Our simulations show the hazards of failing to consider the estimand being sought, particularly when adjusting for total energy intake. In both the isocaloric and equal-mass models of meat compared with fish, a positive effect was observed. However, when the comparison was with cereal, dairy, fish, nuts, and miscellaneous foods, the effect differed depending on whether the substitution concerned calories or grams. Unfortunately, simply adjusting for total energy intake does not guarantee the substitution is isocaloric; the dietary components must also be measured and analyzed in calories to ensure the data are compositional. Where different units are used, the relation between the components and the total is no longer compositional, resulting in an obscure estimand that may be extremely misleading. Our “mixed unit” leave-one-out models of meat and other food components in grams that adjusted for total energy intake returned ambiguous coefficients with opposite sign to the appropriate equal-mass estimands and, on 1 occasion, the appropriate isocaloric estimand. We were unable to establish a firm pattern or meaning for these estimands and therefore advise that the coefficients from “mixed unit” leave-one-out models be treated with caution.

Even where specified appropriately, we have shown that both the “same unit” leave-one-out and energy partition models are prone to bias when the substitution involves ≥2 components. We have previously shown that the standard model for energy intake adjustment, which adjusts for total energy, does not robustly estimate the average relative causal effect owing to “composite variable bias” (14). This occurs because different components with different effects are combined into a single “total energy intake” variable that captures an imperfect average of the individual components. As an extension of the standard model, the leave-one-out model suffers the same problem with substitutions involving >1 component, but the energy partition model also suffers the same issue where composite terms, such as “remaining energy intake,” are used. Where the model includes all components, this problem is mitigated because the weighted average effect can be calculated from each part. However, even this approach is not guaranteed to produce a perfect weighted average because all variables, to a greater or lesser extent, are composites of smaller parts. This explains why none of the food models returned the same estimate as the derived weighted average effect; every food contained multiple macronutrient components with different effects.

### Implications and recommendations

Substitution effects include ≥2 variables in the exposure regime, making it particularly necessary to ensure the estimand is well-defined. We recommend that all studies seeking substitution effects should clearly state their target estimand and justify the adjustment strategy accordingly; directed acyclic graphs can be particularly useful in this regard (24).

What constitutes a well-defined intervention in experimental research and public health practice may differ from what constitutes a well-defined estimand in observational data. In experimental and interventional contexts, greater focus is typically given to food groups or portions because these represent the units of consumption. Food groups or portions may equally be examined in observational data, but their composite nature means that the estimated effects may be less consistent than for macronutrient variables. We encourage researchers to consider which units are most appropriate for their specific interests and recognize the trade-offs involved.

Wherever ≥2 components are involved in the substitution, there is scope for composite variable bias unless the individual effects are estimated and combined using an all-components approach. Because the leave-one-out approach is more common in the literature than the energy partition model (16–20), this raises concerns about the validity of existing estimates that involve more complex substitutions.

Imperfect substitution models may nevertheless represent less of a concern than inadvertent substitution models that arise from naïve adjustments for total energy intake. Total energy intake is routinely included in nutrition epidemiology models for a variety of reasons (8). Unfortunately, if the other model variables are not carefully selected with a specific estimand in mind, the model may estimate inadvertent or obscure causal effects. Where isocaloric substitution effects are desired, all dietary variables must be measured and analyzed in calorie units, regardless of whether the leave-one-out or energy partition model is used and regardless of whether the variables are macronutrients, foods in grams, or foods in portions. Equal-mass substitutions may also be estimated using either approach by including mass variables whether in grams or portions; for the leave-one-out model this will require adjusting for total food in grams or portions, respectively.

These findings have clear implications, not just for the conduct of future research, but for the appraisal and synthesis of existing studies. Meta-analyses of dietary exposures rarely separate studies based on their modeling strategy, making their results difficult to interpret. Where estimates from different estimands have been combined, the results may be misleading or meaningless. Relative causal effects are particularly difficult to compare or summarize because it is extremely common to adjust for several components in addition to total energy intake, creating unique substitution effects (16–20). Studies that have considered ≥1 food variables in grams while adjusting for total energy may contribute obscure estimates with opposite signs to those estimated from appropriate isocaloric or equal-mass models. We believe this is likely to explain some of the apparent heterogeneity in effect estimates between studies, and we recommend that meta-analyses should only pool estimates of the same estimand.

### Limitations and caveats

Our study used simulated data that oversimplify reality. Although informed by real data, our primary aim was to demonstrate the methodological topics at hand; the effects therefore should not be interpreted as true effects. All variables were simulated as multivariate normal, with no measurement error or interactions, which does not reflect reality. All variables were transformed toward observed means (23), but SDs had to be reduced and some negative and/or biologically implausible values were simulated.

We did not simulate or examine the influence of measurement error. Although important, the issues with measurement error are distinct from those presented in the current article. Similarly, we did not simulate or examine the influence of confounding. Although confounding will be ubiquitous in practice, there is no specific relevance to substitution modeling beyond what we have shown previously (14). Both the leave-one-out and energy partition models will be equally robust to confounding provided the substitution involves only 1 component; but residual confounding may arise where ≥2 components are unadjusted (14).

### Conclusion

The “leave-one-out” and energy partition models perform equally well when estimating relative effects that involve substituting a single exposure with a single dietary component. Where more components are involved, both approaches may return biased estimates. Regardless of the approach, substitution models need to be constructed from clearly defined causal effect estimands. The practice of examining foods measured in grams or portions while adjusting for total energy intake is likely to deliver obscure relative effect estimands with unclear inter-pretations.

## Supplementary Material

nqac188_Supplemental_File

## Data Availability

Analytic code, which includes data simulations, is publicly and freely available without restriction at https://github.com/georgiatomova/nutrition-substitution.

## Notes

Author disclosures: PWGT and MSG are both directors of Causal Insights Ltd, which sells causal inference research and training; the company and the authors may therefore benefit from any study that demonstrates the value of causal inference methods.

GDT was supported by PhD funding from The Alan Turing Institute (EP/N510129/1). The Alan Turing Institute had no involvement in the study design; data simulation, analysis, and interpretation; report writing; and restrictions on the submission of the report for publication.

Supplemental Tables 1 and 2 and Supplemental Methods are available from the “Supplementary data” link in the online posting of the article and from the same link in the online table of contents at https://academic.oup.com/ajcn/.
