# Supplementary material for: Theory and performance of substitution models for estimating relative causal effects in nutritional epidemiology
Source: Am J Clin Nutr. 2022 Oct 13;116(5):1379–88. doi: 10.1093/ajcn/nqac188 (PMC9630885; doi:10.1093/ajcn/nqac188)
Supplement: nqac188_Supplemental_File [file nqac188_supplemental_file.docx]

*Tomova et al.* Theory and performance of substitution models for estimating relative causal effects in nutritional epidemiology.

# Supplementary materials

## Supplementary Table 1.

| Variable | Mean (SD) |
| --- | --- |
|  |  |
| Fasting plasma glucose, *mg/dL* | 80 (25) |
|  |  |
| Sugars, *kcal* | 250 (125) |
| Carbohydrates, *kcal* | 900 (315) |
| Fiber, *kcal* | 50 (20) |
| Saturated fat, *kcal* | 200 (90) |
| Unsaturated fat, *kcal* | 400 (200) |
| Protein, *kcal* | 300 (105) |
| Alcohol, *kcal* | 100 (50) |
|  |  |

Target mean and standard deviation values of the variables in the simulated data.

## Supplementary Table 2.

Proportions of macronutrients allocated to each food group in the simulated data.

| Macronutrients → | Protein | Sat fat | Unsat fat | Carbs | Sugars | Fiber | Alcohol |
| --- | --- | --- | --- | --- | --- | --- | --- |
| Food groups ↓ |  |  |  |  |  |  |  |
|  |  |  |  |  |  |  |  |
| Cereal | 0.229 | 0.209 | 0.209 | 0.449 | 0.240 | 0.396 | 0.000 |
| Dairy | 0.173 | 0.237 | 0.126 | 0.054 | 0.064 | 0.011 | 0.000 |
| Meat | 0.357 | 0.246 | 0.234 | 0.063 | 0.043 | 0.125 | 0.000 |
| Fish | 0.073 | 0.032 | 0.049 | 0.011 | 0.000 | 0.023 | 0.000 |
| Fruit & Veg | 0.091 | 0.073 | 0.135 | 0.199 | 0.032 | 0.371 | 0.000 |
| Nuts | 0.011 | 0.022 | 0.038 | 0.000 | 0.000 | 0.000 | 0.000 |
| Alcoholic Beverages | 0.000 | 0.000 | 0.000 | 0.031 | 0.092 | 0.000 | 1.000 |
| Non-alcoholic beverages | 0.011 | 0.000 | 0.000 | 0.072 | 0.228 | 0.000 | 0.000 |
| Miscellaneous | 0.054 | 0.181 | 0.209 | 0.121 | 0.301 | 0.075 | 0.000 |
| *Total* | 1.000 | 1.000 | 1.000 | 1.000 | 1.000 | 1.000 | 1.000 |
|  |  |  |  |  |  |  |  |

## Supplementary Methods

### Macronutrient models

#### Relative causal effect of sugars instead of protein

This estimand ($R_{1}$) is the joint effect of increasing intake of sugars and decreasing intake of protein, while keeping total energy intake unchanged.

##### ‘Leave-one-out’ model

$\hat{FPG}=\hat{a_{0}}+\hat{a_{1}}sugars+ \hat{a_{2}}carbs+\hat{a_{3}}unsat fat+\hat{a_{4}}sat fat+\hat{a_{5}}fiber+\hat{a_{6}}alcohol+\hat{a_{7}}TE+\varepsilon$

This model includes the exposure (sugars), total energy ($TE$), and all components *except* the substituting component (protein). The coefficient $\hat{a_{1}}$directly estimates $R_{1}$.

##### (Comprehensive) energy partition model and all-components model

$\hat{FPG}=\hat{b_{0}}+\hat{b_{1}}sugars+\hat{b_{2}}protein+\hat{b_{3}}carbs+\hat{b_{4}}unsat fat+\hat{b_{5}}sat fat+\hat{b_{6}}fiber+ \hat{b_{7}}alcohol+\varepsilon$

When the energy partition model includes all components, it becomes the all-components model. This model includes individual terms for the exposure (sugars), the substituting component (protein), and all other dietary components. $R_{1}$ may be estimated from the difference between the coefficients for the exposure ($b_{1}$) and the substituting component ($b_{2}$), i.e., $\hat{R_{1}}=\hat{b_{1}}- \hat{b_{2}}$.

#### (Inadvertent) relative causal effect of sugars instead of protein, total fat, and fiber

This (inadvertent) estimand ($R_{2}$) is the joint effect of increasing intake of sugars and decreasing intake of protein, total fat, and fiber, while keeping total energy intake unchanged.

##### Inadvertent ‘leave-one-out’ model

$\hat{FPG}=\hat{c_{0}}+\hat{c_{1}}sugars+\hat{c_{2}}TE+\hat{c_{3}}carbs+\hat{c_{4}}alcohol+\varepsilon$

This model includes the exposure (sugar), total energy, carbohydrates, and alcohol. A substitution is formed with the omitted components (protein, total fat, and fiber). The coefficient $\hat{c_{1}}$directly estimates $R_{2}$.

##### All-components model

$R_{2}$ can be estimated using model 1.2 from the difference between the coefficient for the exposure (sugar, $\hat{b_{1}}$) and a weighted average ($\hat{b_{2,4,5,6}}$) of the coefficients for the substituting components (protein, $\hat{b_{2}}$, unsaturated fat, $\hat{b_{4}}$, saturated fat, $\hat{b_{5}}$, and fiber $\hat{b_{6}}$); i.e., $\hat{R_{2}}= \hat{b_{1}}-\hat{b_{2,4,5,6}}$ where $\hat{b_{2,4,5,6}}=\sum iw_{i}b_{i}$ and $w_{i}$ is the proportion of energy intake from all substituting components contributed by each $i=\{2,4,5,6\}$).

#### Average relative causal effect

This estimand ($R_{3}$) is the joint effect of increasing intake of sugars and decreasing intake of all other dietary components, while keeping total energy intake unchanged.

##### ‘Leave-one-out’ model

$\hat{FPG}= \hat{d_{0}}+\hat{d_{1}}sugars+\hat{d_{2}}TE+\varepsilon$

This model includes the exposure (sugar) and total energy only; all other components are 'left out' to form the substitution. The coefficient $\hat{d_{1}}$ directly estimates $R_{3}$.

##### (Simple) energy partition model

$\hat{FPG}=\hat{f_{0}}+\hat{f_{1}}sugars+\hat{f_{2}}RE+\varepsilon$

This model includes the exposure (sugar) and a summary term for all remaining energy ($RE$) only. $R_{3}$ is estimated from the difference between the the exposure ($\hat{f_{1}}$) and remaining energy ($\hat{f_{2}}$) terms , i.e., $\hat{R_{3}}=\hat{f_{1}}- \hat{f_{2}}$.

##### All-components model

$R_{3}$ can be estimated using model 1.2 from the difference between the coefficient for the exposure (sugar, $b_{1}$) and a weighted average ($b_{2:7}$) of the coefficients for all other components (protein, $b_{2}$, carbohydrates, $b_{3}$ unsaturated fat, $b_{4}$, saturated fat, $b_{5}$, fiber, $b_{6}$, and alcohol, $b_{7}$); i.e., $\hat{R_{2}}= \hat{b_{1}}-\hat{b_{2:7}}$ where $\hat{b_{2:7}}=\sum iw_{i}b_{i}$ and $w_{i}$ is the proportion of the remaining energy intake contributed by each component $i=\{2,3,4,5,6,7\}$)..

### Food group models (in calories)

#### Relative causal effect of meat instead of fish (in calories)

This estimand ($R_{4}$) is the joint effect of increasing intake of meat (in calories) and decreasing intake of fish (in calories), while keeping total energy intake unchanged.

##### 'Leave-one-out' model

$\hat{FPG}=\hat{g_{0}}+\hat{g_{1}}meat+\hat{g_{2}}cereal+\hat{g_{3}}dairy+\hat{g_{4}}fruit veg+\hat{g_{5}}nuts+\hat{g_{6}}alc bev+ \hat{g_{7}}nonalc bev+ \hat{g_{8}}misc+\hat{g_{9}}TE+\varepsilon$

This model includes the exposure (meat), total energy, and all components *except* the substituting component (fish). All variables are measured in calories. The coefficient $\hat{g_{1}}$directly estimates $R_{4}$.

##### All-components model

$\hat{FPG}=\hat{h_{0}}+\hat{h_{1}}meat+\hat{h_{2}}cereal+\hat{h_{3}}dairy+\hat{h_{4}}fish+\hat{h_{5}}fruit veg+\hat{h_{6}}nuts+ \hat{h_{7}}alc bev+ \hat{h_{8}}nonalc bev+\hat{h_{9}}misc+\varepsilon$

This all-components includes individual terms for the exposure (meat), the substituting component (fish), and all other dietary components. All variables are measured in calories. $R_{4}$ may be estimated from the difference between the coefficients for the exposure ($h_{1}$) and the substituting component ($h_{4}$), i.e., $\hat{R_{4}}=\hat{h_{1}}- \hat{h_{4}}$.

#### (Inadvertent) relative causal effect of meat instead of cereal, dairy, fish, nuts, and miscellaneous food (in calories)

This (inadvertent) estimand ($R_{5}$) is the joint effect of increasing intake of meat (in calories) and decreasing intake of cereal, dairy, fish, nuts, and miscellaneous foods (in calories), while keeping total energy intake unchanged.

##### (Inadvertent) 'leave-one-out' model

$\hat{FPG}=\hat{i_{0}}+\hat{i_{1}}meat+\hat{i_{2}}fruit veg+\hat{i_{3}}alc bev+\hat{i_{4}}nonalc bev+\hat{i_{5}}TE+\varepsilon$

This model includes the exposure (sugar), total energy, carbohydrates, and alcohol. All variables are measured in calories. The substitution is made with the omitted components (protein, total fat, and fiber). The coefficient $\hat{l_{1}}$directly estimates $R_{5}$.

##### All-components model

$R_{5}$ can be estimated using model 4.2 from the difference between the coefficient for the exposure (meat, $h_{1}$) and a weighted average ($\hat{h_{2,3,4,6,9}}$) of the coefficients for the substituting components (cereal, $\hat{h_{2}}$, dairy, $\hat{h_{3}}$, fish, $\hat{h_{4}}$, nuts, $\hat{h_{6}}$, and miscellaneous foods ($\hat{h_{9}}$); i.e., $\hat{R_{5}}= \hat{h_{1}}-\hat{h_{2,3,4,6,9}}$ where $\hat{h_{2,3,4,6,9}}=\sum iw_{i}h_{i}$ and $w_{i}$ is the proportion of energy intake from all the substituting components contributed by each $i=\left\{ 2,3,4,6,9 \right\}$.

#### Average relative causal effect of meat (in calories)

This estimand ($R_{6}$) is the joint effect of increasing intake of meat (in calories) and decreasing intake of all other foods (in calories), while keeping total energy intake unchanged.

##### 'Leave-one-out' model

$\hat{FPG}=\hat{j_{0}}+\hat{j_{1}}meat+\hat{j_{2}}TE+\varepsilon$

This model includes the exposure (meat) and total energy only; all other components are 'left out' to form the substitution. Both variables are measured in calories. The coefficient $\hat{j_{1}}$ directly estimates $R_{6}$.

##### (Simple) energy partition model

$\hat{FPG}=\hat{k_{0}}+\hat{k_{1}}meat+\hat{k_{2}}RE+\varepsilon$

This model includes the exposure (meat, in calories) and a summary term for all remaining energy (in calories) only. $R_{6}$ is estimated from the difference between the exposure ($\hat{k_{1}}$) and remaining energy ($\hat{k_{2}}$) terms, i.e., $\hat{R_{6}}=\hat{k_{1}}- \hat{k_{2}}$.

##### All-components model

$R_{6}$ can be estimated using model 4.2 from the difference between the coefficient for the exposure (meat, $\hat{h_{1}}$) and a weighted average ($\hat{h_{2:9}}$) of the coefficients for all other components (cereal, $\hat{h_{2}}$, dairy, $\hat{h_{3}}$, fish, $\hat{h_{4}}$, fruit and veg, $\hat{h_{5}}$, nuts, $\hat{h_{6}}$, alcohol beverages, $\hat{h_{7}}$, non-alcoholic beverages, $\hat{h_{8}}$, and miscellaneous foods ($\hat{h_{9}}$); i.e., $\hat{R_{5}}= \hat{h_{1}}-\hat{h_{2:9}}$ where $\hat{h_{2:9}}=\sum iw_{i}h_{i}$ and $w_{i}$ is the proportion of the remaining energy intake contributed by each $i=\left\{ 2,3,4,5,6,7,8,9 \right\}$.

### Food group models (in grams)

#### Relative causal effect of meat instead of fish (in grams)

This estimand ($R_{7}$) is the joint effect of increasing the intake of meat (in grams) and decreasing the intake of fish (in grams), while keeping total food intake or total energy intake unchanged.

##### 'Leave-one-out' model, adjusting for total food intake

$\hat{FPG}=\hat{l_{0}}+\hat{l_{1}}meat+\hat{l_{2}}cereal+\hat{l_{3}}dairy+\hat{l_{4}}fruit veg+\hat{l_{5}}nuts+\hat{l_{6}}alc bev+ \hat{l_{7}}nonalc bev+ \hat{l_{8}}misc+\hat{l_{9}}TF+\varepsilon$

This model includes the exposure (meat), total *food* intake ($TF$), and all components *except* the substituting component (fish). All variables are in grams. The coefficient $\hat{l_{1}}$directly estimates $R_{7}$.

##### 'Leave-one-out' model, adjusting for total energy intake

$\hat{FPG}=\hat{m_{0}}+\hat{m_{1}}meat+\hat{m_{2}}cereal+\hat{m_{3}}dairy+\hat{m_{4}}fruit veg+\hat{m_{5}}nuts+\hat{m_{6}}alc bev+ \hat{m_{7}}nonalc bev+ \hat{m_{8}}misc+\hat{m_{9}}TE+\varepsilon$

This model includes the exposure (meat), total *energy*, and all components *except* the substituting component (fish). All variables are measured in grams except total energy. The coefficient $\hat{m_{1}}$evaluates an obscure estimand.

##### All-components model

$\hat{FPG}=\hat{n_{0}}+\hat{n_{1}}meat+\hat{n_{2}}cereal+\hat{n_{3}}dairy+\hat{n_{4}}fish+\hat{n_{5}}fruit veg+\hat{n_{6}}nuts+ \hat{n_{7}}alc bev+ \hat{n_{8}}nonalc bev+\hat{n_{9}}misc+\varepsilon$

This all-components includes individual terms for the exposure (meat), the substituting component (fish), and all other dietary components. All variables are measured in grams. $R_{7}$ may be estimated from the difference between the coefficients for the exposure ($n_{1}$) and the substituting component ($n_{4}$), i.e., $\hat{R_{7}}=\hat{n_{1}}- \hat{n_{4}}$.

#### (Inadvertent) relative causal effect of meat instead of cereal, dairy, fish, nuts, and miscellaneous (in grams)

This estimand ($R_{8}$) is the joint effect of increasing intake of meat (in grams) and decreasing intake of cereal, dairy, fish, nuts, and miscellaneous (in grams), while keeping total food intake or total energy intake unchanged.

##### (Inadvertent) 'leave-one-out' model, adjusting for total food intake

$\hat{FPG}=\hat{o_{0}}+\hat{o_{1}}meat+\hat{o_{2}}fruit veg+\hat{o_{3}}alc bev+\hat{o_{4}}nonalc bev+\hat{o_{5}}TF+\varepsilon$

This model includes the exposure (meat), total *food* intake, fruit and veg, alcohol beverages, and non-alcoholic beverages. All variables are measured in grams. The substitution is made with the omitted components (cereal, dairy, fish, nuts, and miscellaneous foods). The coefficient $\hat{o_{1}}$directly estimates $R_{8}$.

##### 'Leave-one-out' model, adjusting for total energy intake

$\hat{p_{0}}+\hat{p_{1}}meat+\hat{p_{2}}fruit veg+\hat{p_{3}}alc bev+\hat{p_{4}}nonalc bev+\hat{p_{5}}TE+\varepsilon$

This model includes the exposure (meat), total *energy*, fruit and veg, alcohol beverages, and non-alcoholic beverages. All variables are measured in grams except total energy. The coefficient $\hat{p_{1}}$evaluates an obscure estimand.

##### All-components model

$R_{5}$ can be estimated using model 7.3 from the difference between the coefficient for the exposure (meat, $\hat{n_{1}}$) and a weighted average ($\hat{n_{2,3,4,6,9}}$) of the coefficients for the substituting components (cereal, $\hat{n_{2}}$, dairy, $\hat{n_{3}}$, fish, $\hat{n_{4}}$, nuts, $\hat{n_{6}}$, and miscellaneous foods ($\hat{n_{9}}$); i.e., $\hat{R_{8}}= \hat{n_{1}}-\hat{n_{2,3,4,6,9}}$ where $\hat{n_{2,3,4,6,9}}=\sum iw_{i}n_{i}$ and $w_{i}$ is the proportion of energy intake from all the substituting components contributed by each $i=\left\{ 2,3,4,6,9 \right\}$.

#### Average relative causal effect of meat (in grams)

This estimand ($R_{9}$) is the joint effect of increasing intake of meat (in grams) and decreasing intake of all other foods (in grams), while keeping total food intake or total energy intake unchanged.

##### 'Leave-one-out' model, adjusting for total food intake

$\hat{q_{0}}+\hat{q_{1}}meat+\hat{q_{2}}TF+\varepsilon$

This model includes the exposure (meat) and total *food* intake only; all other components are 'left out' to form the substitution. Both variables are measured in grams. The coefficient $\hat{q_{1}}$ directly estimates $R_{9}$.

##### 'Leave-one-out' model, adjusting for total energy intake

$\hat{r_{0}}+\hat{r_{1}}meat+\hat{r_{2}}TE+\varepsilon$

This model includes the exposure (meat, in grams) and total *energy* (in calories) only. The coefficient $\hat{r_{1}}$evaluates an obscure estimand.

##### (Simple) energy partition model

$\hat{s_{0}}+\hat{s_{1}}meat+\hat{s_{2}}RF+\varepsilon$

This model includes the exposure (meat, in grams) and a summary term for all remaining food intake (in grams) only. $R_{9}$ is estimated from the difference between the exposure ($\hat{s_{1}}$) and remaining energy ($\hat{s_{2}}$) terms, i.e., $\hat{R_{9}}=\hat{s_{1}}- \hat{s_{2}}$.

##### All-components model

$R_{9}$ can be estimated using model 7.3 from the difference between the coefficient for the exposure (meat, $\hat{n_{1}}$) and a weighted average ($\hat{n_{2:9}}$) of the coefficients for all other components (cereal, $\hat{n_{2}}$, dairy, $\hat{n_{3}}$, fish, $\hat{n_{4}}$, fruit and veg, $\hat{n_{5}}$, nuts, $\hat{n_{6}}$, alcohol beverages, $\hat{n_{7}}$, non-alcoholic beverages, $\hat{n_{8}}$, and miscellaneous foods ($\hat{n_{9}}$); i.e., $\hat{R_{9}}= \hat{n_{1}}-\hat{n_{2:9}}$ where $\hat{n_{2:9}}=\sum iw_{i}n_{i}$ and $w_{i}$ is the proportion of the remaining food intake contributed by each $i=\left\{ 2,3,4,5,6,7,8,9 \right\}$.
